# Transcriptomic analysis of KSHV-infected primary oral fibroblasts: The role of interferon-induced genes in the latency of oncogenic virus

**DOI:** 10.18632/oncotarget.9720

**Published:** 2016-05-30

**Authors:** Lu Dai, Lihua Bai, Zhen Lin, Jing Qiao, Liang Yang, Erik K. Flemington, Jovanny Zabaleta, Zhiqiang Qin

**Affiliations:** ^1^ Department of Oncology, East Hospital, Tongji University School of Medicine, Shanghai, 200120, China; ^2^ Research Center for Translational Medicine and Key Laboratory of Arrhythmias, East Hospital, Tongji University School of Medicine, Shanghai, 200120, China; ^3^ Departments of Microbiology/Immunology/Parasitology, Louisiana State University Health Sciences Center, Louisiana Cancer Research Center, New Orleans, LA, 70112, USA; ^4^ Department of Medicine, Louisiana State University Health Sciences Center, Louisiana Cancer Research Center, New Orleans, LA, 70112, USA; ^5^ Department of Pediatrics, East Hospital, Tongji University School of Medicine, Shanghai, 200120, China; ^6^ Department of Pathology, Tulane University Health Sciences Center, Tulane Cancer Center, New Orleans, LA, 70112, USA; ^7^ Singapore Centre for Environmental Life Sciences Engineering (SCELSE), Nanyang Technological University, Singapore, 637551, Singapore; ^8^ Department of Pediatrics, Louisiana State University Health Sciences Center, Louisiana Cancer Research Center, New Orleans, LA, 70112, USA

**Keywords:** KSHV, interferon, oral fibroblast, viral oncogenesis

## Abstract

The Kaposi sarcoma-associated herpesvirus (KSHV) is the causative agent of Kaposi sarcoma (KS), the most common HIV/AIDS-associated tumor worldwide. Involvement of the oral cavity portends a poor prognosis for patients with KS, but the mechanisms for KSHV regulation of the oral tumor microenvironment are largely unknown. Infiltrating fibroblasts are found within KS lesions, and KSHV can establish latent infection within human primary fibroblasts *in vitro* and *in vivo*, but contributions for KSHV-infected fibroblasts to the KS microenvironment have not been previously characterized. In the present study, we used Illumina microarray to determine global gene expression changes in KSHV-infected primary human oral fibroblasts (PDLF and HGF). Among significantly altered candidates, we found that a series of interferon-induced genes were strongly up-regulated in these KSHV-infected oral cells. Interestingly, some of these genes in particular *ISG15* and *ISG20* are required for maintenance of virus latency through regulation of specific KSHV microRNAs. Our data indicate that oral fibroblasts may represent one important host cellular defense component against viral infection, as well as acting as a reservoir for herpesvirus lifelong infection in the oral cavity.

## INTRODUCTION

Kaposi sarcoma-associated herpesvirus (KSHV) is one of the most common etiologic agents for cancers arising in the setting of immune suppression, including Kaposi sarcoma (KS)—the most common HIV/AIDS-associated tumor worldwide and a leading cause of morbidity and mortality in this population [1]. Oral involvement occurs in a substantial proportion of patients with KS [2]. Published literatures suggest that KSHV dissemination within and from the oral cavity are critical factors for KSHV infection and oral KS progression in HIV-infected patients [3–7]. Person-to-person transmission of KSHV is thought to occur primarily through exchange of oropharyngeal secretions [3, 4], and epidemiologic data indicate that sexual practices involving contact with the oral cavity may promote KSHV transmission [5]. Furthermore, people have found that combination antiretroviral therapy (cART) cannot reduce KSHV replication within the oropharynx [3, 5] or KSHV transmission [7].

Oral KS lesions usually display higher KSHV viral loads and may portend more ominous prognoses relative to KS in other anatomic locations [8, 9]. We recently reported that KSHV successfully established latent infection in primary human gingival fibroblasts (HGF) or periodontal ligament fibroblasts (PDLF) *in vitro*, and virus *de novo* infection induced a tumor-associated fibroblast (TAF)-like phenotype within these cells [10]. Others also demonstrated that fibroblasts represented an imporant component within KS lesions and supported *de novo* KSHV infection [11, 12]. In addition, we recently reported that some pathogen-associated molecular patterns (PAMPs) molecules from periodontal pathogenic bacteria increased KSHV entry and subsequent viral latent gene expression within oral fibroblasts [13]. Despite this knowledge, the global altered gene expression profile in KSHV-infected oral fibroblasts has never been reported. KSHV needs to manipulate a number of host genes to facilitate the establishment of lifelong latent infection. In the current study, we used Illumina microarray to assess the altered gene profile in KSHV-infected PDLF and HGF relative to unifected mock cells. We found that the expression of various gene sets are significantly changed in virus-infected cells. In particular, KSHV *de novo* infection strongly up-regulates a series of interferon-induced gene in these oral cells, which are closely related to the maintenancy of virus latency.

## RESULTS AND DISCUSSION

### Microarray analysis of the global gene expression changes in *KSHV*-infected primary oral fibroblasts

**Figure 1:**
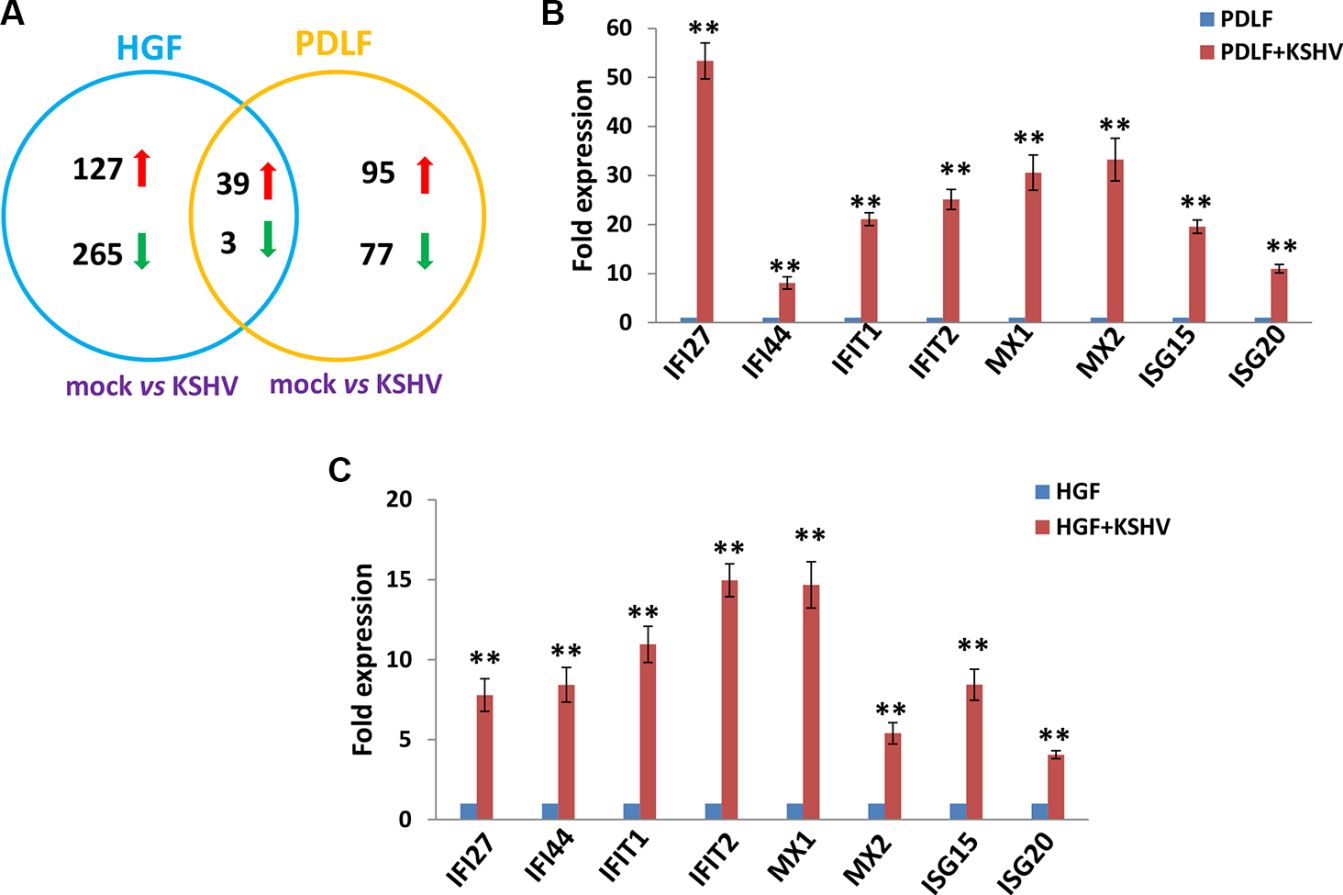
Intersection analysis and experimental validation of gene profile alterations in KSHV-infected primary oral fibroblast cells (**A**) The HumanHT-12 v4 Expression BeadChip (Illumina) was used to detect alterations in gene profile in PDLF or HGF cells infected by KSHV (MOI~10, *vs* respective mock cells). Intersection analysis of significantly altered genes (up/down ≥ 2 fold and *p* < 0.05) was performed using the Illumina GenomeStudio Software. (**B**–**C**) The transcriptional levels of 8 selected ‘common’ candidate genes that were up-regulated in both sets of microarray data were validated by using qRT-PCR. Error bars represent the S.E.M. for 3 independent experiments. ** = *p* < 0.01 (*vs* PDLF or HGF).

**Figure 2:**
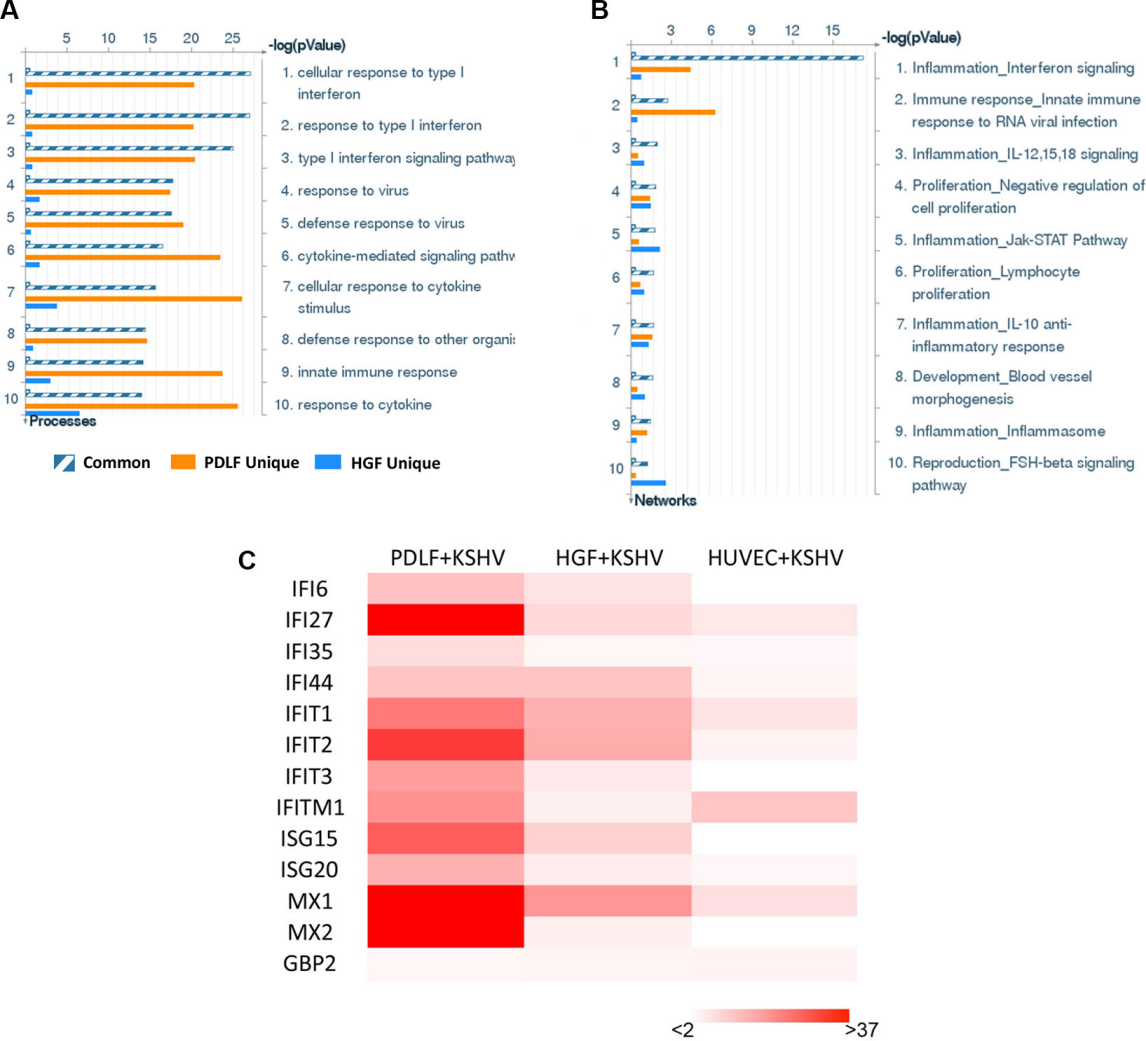
The enrichment analysis of gene profile alterations in KSHV-infected primary oral fibroblast cells (**A**–**B**) The enrichment analysis of gene profile significantly altered (up/down ≥ 2 fold and *p* < 0.05) in KSHV-infected PDLF or HGF cells (*vs* mock cells) was performed using the Metacore Software (Thompson Reuters) Modules: Gene Ontology Processes (A) and Process Networks (B). (**C**) Heat map of interferon-induced genes signature altered in KSHV-infected PDLF, HGF and HUVEC cells (*vs* respective mock cells) was made by using Microsoft Excel 2010.

We first used the HumanHT-12 v4 Expression BeadChip (Illumina), which contains more than 47,000 probes derived from the NCBI RefSeq Release 38 and other sources, to study global gene expression changes altered within KSHV-infected PDLF or HGF cells. We found that in PDLF cells, 134 genes were significantly up-regulated and 80 were down-regulated (≥ 2 fold and *p* < 0.05); in HGF cells, 166 genes were up-regulated and 268 down-regulated (Figure 1A). Intersection analysis indicated that 39 “common” genes were significantly up-regulated and 3 were down-regulated in both cell lines (listed in Table 1). We also performed enrichment analysis of these “common” genes in both cell lines by using the Pathway map, Gene Ontology (GO) Processes and Process Networks modules from Metacore Software (Thompson Reuters) [14]. Our analysis showed that most genes belong to several major cellular function categories, such as cellular response to type I interferon (IFN), inflammatory cytokine production, and other innate immune responses (Figure 2A–2B). The top 2 scored pathway maps (immune response_IFN α/β signaling pathway and immune response_Thymic stromal lymphopoietin [TSLP] signaling pathway) for these “common” genes are shown in Supplementary Figure S1. Interestingly, aberrant TSLP/TSLPR signaling has been associated with a variety of human diseases including asthma, atopic dermatitis, inflammatory bowel disease, eosinophilic esophagitis and acute lymphoblastic leukemia [15], but it has never been reported in KSHV infection and/or related malignancies.

**Table 1:** The “common” genes set altered within KSHV-infected HGF and PDLF cells (*vs* mock cells)

Gene Symbol	Gene Description	PDLF (folds)	HGF (folds)
IFI27	Interferon alpha-inducible protein 27, mitochondrial	57.85	6.07
RSAD2	Radical S-adenosyl methionine domain-containing protein 2	43.99	4.48
MX1	Interferon-induced GTP-binding protein Mx1	36.48	15.76
MX2	Interferon-induced GTP-binding protein Mx2	36.48	3.26
IFIT2	Interferon-induced protein with tetratricopeptide repeats 2	28.42	12.6
ISG15	Ubiquitin-like protein ISG15	23.67	7.46
IFIT1	Interferon-induced protein with tetratricopeptide repeats 1	19.65	11.95
IFITM1	Interferon-induced transmembrane protein 1	16.44	3.16
HERC6	Probable E3 ubiquitin-protein ligase HERC6	14.97	4.47
IFIT3	Interferon-induced protein with tetratricopeptide repeats 3	14.77	3.84
ISG20	Interferon-stimulated gene 20 kDa protein	12.03	3.38
IFI6	Interferon alpha-inducible protein 6	9.5	4.89
IFI44	Interferon-induced protein 44	9.13	9.24
SAMD9	Sterile alpha motif domain-containing protein 9	8.07	3.16
EPSTI1	Epithelial-stromal interaction protein 1	7.02	3.53
RARRES3	Retinoic acid receptor responder protein 3	5.71	2.19
IFI35	Interferon-induced 35 kDa protein	5.64	2.35
XAF1	XIAP-associated factor 1	5.32	2.22
DDX58	Probable ATP-dependent RNA helicase DDX58	4.85	2.19
SAMD9L	Sterile alpha motif domain-containing protein 9-like	4.7	3.08
STAT1	Signal transducer and activator of transcription 1-alpha/beta	4.6	2.29
PARP12	Poly [ADP-ribose] polymerase 12	4.38	2.06
DDX60	Probable ATP-dependent RNA helicase DDX60	4.04	2.25
MYPN	Myopalladin	3.26	2.3
IL12A	Interleukin-12 subunit alpha	3.03	5.81
PSG7	Putative pregnancy-specific beta-1-glycoprotein 7	3.02	4.46
COL4A1	Collagen alpha-1(IV) chain	2.72	2.56
PSG1	Pregnancy-specific beta-1-glycoprotein 1	2.67	4.46
PSG2	Pregnancy-specific beta-1-glycoprotein 2	2.67	6.05
ANO3	Anoctamin-3	2.54	6.2
IL7R	Interleukin-7 receptor subunit alpha	2.48	6.46
NR2C1	Nuclear receptor subfamily 2 group C member 1	2.39	6.34
PSG4	Pregnancy-specific beta-1-glycoprotein 4	2.39	6.34
GBP2	Interferon-induced guanylate-binding protein 2	2.38	2.44
KRTAP1-1	Keratin-associated protein 1-1	2.24	2.48
VEGFC	Vascular endothelial growth factor C	2.23	3.04
MT1M	Metallothionein-1M	2.21	2.05
KRT34	Keratin, type I cuticular Ha4	2.1	9.24
PSME2	Proteasome activator complex subunit 2	2.02	2.19
RCAN2	Calcipressin-2	0.47	0.41
CEMIP	Cell migration-inducing and hyaluronan-binding protein	0.4	0.07
ATP8B4	Probable phospholipid-transporting ATPase IM	0.38	0.25

### *IFN*-induced genes are highly up-regulated in *KSHV*-infected primary oral fibroblasts

Among these “common” genes, we noticed that a series of IFN-induced genes were strongly up-regulated in KSHV-infected primary oral fibroblasts (Table 1). We next selected 8 IFN-induced genes from Table 1 for validation of their transcriptional changes by using qRT-PCR analysis. Our results indicated that all of these genes (*IFI27*, *IFI44*, *IFIT1*, *IFIT2*, *MX1*, *MX2*, *ISG15* and *ISG20*) were significantly up-regulated in a manner comparable to those found in the microarray data (Figure 1B–1C), demonstrating the credibility of our microarray analysis. Interestingly, when compared to the microarray data in KSHV-infected primary endothelial cells (HUVEC) we recently published [16], we found that the up-regulation of IFN-induced genes were much stronger in KSHV-infected PDLF/HGF than those in KSHV-infected HUVEC cells (Figure 2C). Production of IFN, in particular type I IFN is one of the most important host anti-viral immune responses, which can induce an anti-viral transcriptional program, producing proteins that cooperate to inhibit the spread of infection. Therefore, our data indicate that oral fibroblasts may represent an important cellular resource for type I IFN production during KSHV infection stimulus in the microenvironment of oral cavity. However, KSHV has successfully established the escape mechanisms from host immune responses, including the type I IFN response. For example, KSHV encodes 4 viral homologs of cellular interferon regulatory factors (named as vIRF1, −2, −3, and −4) with pleiotropic functions such as evasion of cell death, increased proliferation and evasion of immune responses [17]. For example, previous data have demonstrated that the expression of vIRF1 and vIRF2 can inhibit increases in IFN-β mediated by Toll-like receptor 3 (TLR3) [18]. In addition to their immunoregulatory effects, KSHV-encoded vIRFs were also shown to modulate cell growth by targeting the function of the tumor suppressor p53 and enhancing the activity of the c-Myc proto-oncogene [19]. While the KSHV-encoded vIRFs share an ability to block IFN or p53 signaling, each vIRF demonstrates a unique ability to block specific cellular functions [17].

### *IFN*-induced genes, *ISG15* and *ISG20*, are required for maintenance of *KSHV* latency in oral fibroblasts

**Figure 3:**
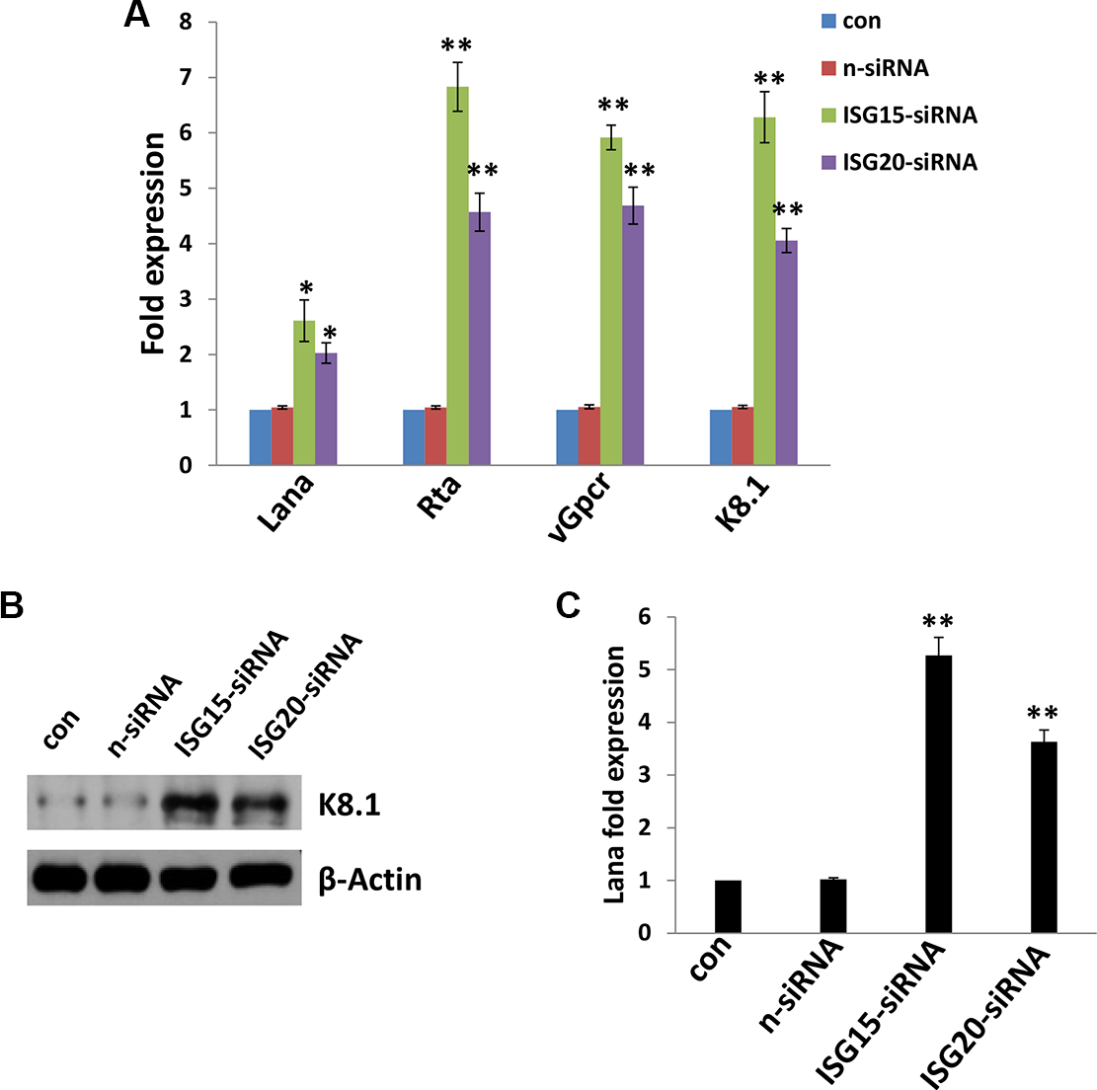
Targeting *ISG15* and/or *ISG20* induces KSHV lytic reactivation from infected primary oral fibroblast cells (**A**–**B**) PDLF were first incubated with purified KSHV (MOI~10) for 2 h, then after 24 h p.i. transfected with either control non-target (n-siRNA), *ISG15*-siRNA or *ISG20*-siRNA for additional 48 h. Viral representative latent (*Lana*) and lytic gene (*Rta*, *vGpcr*, *K8.1*) transcripts were quantified using qRT-PCR. Protein expression was measured by immunoblots. (**C**) Released virions was isolated and purified from supernatant from groups in (A), then used to infect fresh PDLF cells. After 24 h p.i., *Lana* transcripts were quantified using qRT-PCR. Error bars represent the S.E.M for three independent experiments. * = *p* < 0.05, ** = *p* < 0.01 (*vs* n-siRNA group).

Like other herpesviruses, KSHV can establish a lifelong infection in the host, and in more than 90% of infected host cells, the virus exists in a latency stage. Here we found that at least 2 IFN-induced genes, *ISG15* and *ISG20*, are required for maintenance of KSHV latency in oral fibroblasts. Our data indicated that directly targeting *ISG15* or *ISG20* by RNAi significantly caused viral lytic gene (e.g. *Rta*, *vGpcr* and *K8.1*) transcripts from latently-infected PDLF with qRT-PCR analysis (Figure 3A and Supplementary Figure S2). We also confirmed the strong up-regulation of lytic K8.1 protein expression in either *ISG15* or *ISG20* “knock-down” KSHV-infected PDLF by using immunoblots (Figure 3B). Next, we isolated the KSHV virions from conditioned medium of *ISG15* or *ISG20* “knock-down” or control cells, then infected fresh PDLF cells. We found that silencing of either *ISG15* or *ISG20* greatly increased the virion release (there were more *Lana* transcripts in these infected groups compared to controls) (Figure 3C). Interestingly, one very recent study also reported that silencing of *ISG15* in KSHV latently infected iSLK.219 cells resulted in a higher level of virus reactivation and an increase in infectious virus production [20]. They also found that KSHV-encoded vIRF1 protein can inhibit IFN activation in response to viral infection, through interaction with HERC5, an ISG15 E3 ligase, to alter ISG15 modification of cellular proteins [20]. Interestingly, vIRF1 itself was also a target of ISG15 conjugation. KSHV-infected cells exhibited increased ISG15 conjugation upon reactivation from latency in coordination with increased IFN [20].

### *KSHV* microRNAs are involved in viral lytic reactivation caused by silencing of *ISG15* or *ISG20*

**Figure 4:**
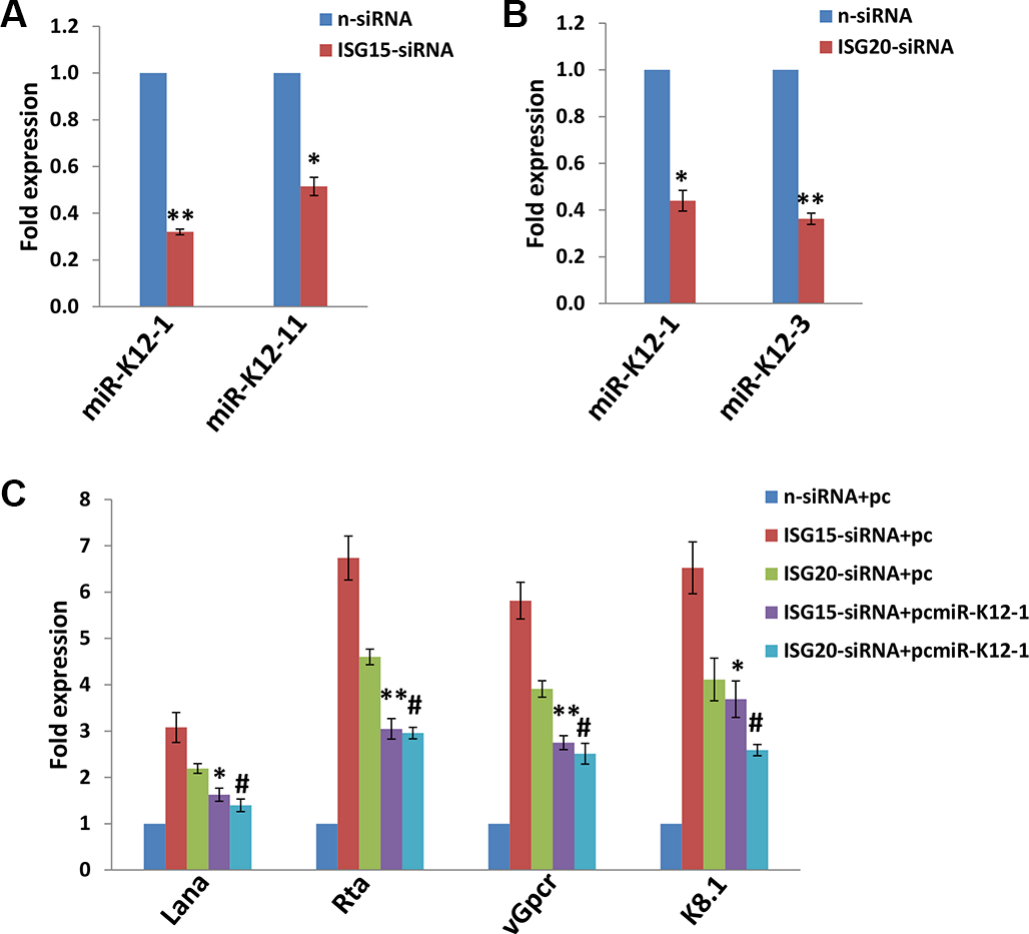
Targeting *ISG15* and/or *ISG20* induces KSHV lytic gene expression through suppression of KSHV microRNAs (**A**–**B**) PDLF were first incubated with purified KSHV (MOI~10) for 2 h, then after 24 h p.i. transfected with either control non-target (n-siRNA), *ISG15*-siRNA or *ISG20*-siRNA for additional 48 h. KSHV microRNA transcripts were quantified using qRT-PCR as described in Methods. (**C**) PDLF were incubated with purified KSHV for 2 h, then transfected with control vector (pc), or vectors encoding miR-K12-1 (pcmiR-K12-1) for additional 24 h. Thereafter, cells were transfected with either control non-target (n-siRNA), *ISG15*-siRNA or *ISG20*-siRNA for additional 48 h. Viral representative latent (*Lana*) and lytic gene (*Rta*, *vGpcr*, *K8.1*) transcripts were quantified using qRT-PCR. Error bars represent the S.E.M for three independent experiments. */# = *p* < 0.05, ** = *p* < 0.01 (*vs ISG15*-siRNA+pc or *ISG20*-siRNA+pc groups, respectively).

KSHV-microRNAs (mostly miR-K12-1, 3, 4, 5, 7, 9 and 11) have been shown to positively or negatively regulate viral latency in a variety of infected host cells, through either directly targeting the viral lytic reactivation activator, Rta [21, 22], or through indirect mechanisms including targeting host factors such as IκBα, nuclear factor I/B (NFIB), Rbl2, BCLAF1 and IKKε [23–27]. By using qRT-PCR screening analysis, we found that silencing of *ISG15* prominently reduced the transcripts of miR-K12-1 and miR-K12-11, while silencing of *ISG20* caused a significant reduction of miR-K12-1 and miR-K12-3 in PDLF cells (Figure 4A–4B). To further confirm the role of specific viral microRNA in *ISG15*- or *ISG20*-mediated virus latency, we used individual recombinant construct encoding miR-K12-1 as described previously [28] to restore its expressional level. We found that this overexpression of miR-K12-1 significantly repressed KSHV lytic gene expression for infected PDLF cells during “knock-down” *ISG15* or *ISG20* with RNAi (Figure 4C). Published data have shown that miR-K12-1 can targets IκBα, an inhibitor of NF-κB complexes, thereby promoting NF-κB-dependent viral latency and cell survival [23]. Our recent data also demonstrate that the NF-κB pathway is important to KSHV-positive lymphoma cell survival and viral latency [29]. Therefore, ongoing work will try to understand the involvement of NF-κB pathway in either *ISG15*- or *ISG20*-mediated virus latency for oral cells.

In summary, we provide for the first time global gene expression profile alterations in KSHV-infected oral fibroblasts by microarray analysis. Among the altered candidates, many interferon-induced genes are strongly up-regulated in these oral cells, while some of them such as *ISG15* and *ISG20* are required for the maintenance of virus latency. Our data indicate that oral fibroblasts may represent one of the important host cellular defense components for anti-viral infection, as well as acting as a reservoir for herpesvirus lifelong infection in the oral cavity.

## MATERIALS AND METHODS

### Cell culture, reagents and infection protocol

Body cavity-based lymphoma cells (BCBL-1, KSHV^+^/EBV^−^) were maintained in RPMI 1640 medium (Gibco) with supplements as described previously [29]. Primary human gingival fibroblasts (HGF) and periodontal ligament fibroblasts (PDLF) were purchased from ScienCell. These cells were maintained in Dulbecco's modified Eagle's medium (DMEM, Mediatech) supplemented with 10% FBS, 10 mM HEPES (pH 7.5), 100 U/mL of penicillin, 100 μg/mL streptomycin, and 0.25 μg/mL amphotericin B. All cells were incubated at 37°C in 5% CO_2_. All experiments were carried out using cells harvested at low (< 20) passages. To obtain KSHV for infection experiments, BCBL-1 cells were incubated with 0.6 mM valproic acid for 6 days, and purified virus was concentrated from culture supernatants and infectious titers were determined as described previously [28].

### Microarray

Total RNA was isolated using Qiagen RNeasy kit (Qiagen), and 500 ng of total RNA was used to synthesize dscDNA. Biotin-labeled RNA was generated using the TargetAmp-Nano Labeling Kit for Illumina Expression BeadChip (Epicentre), according to the manufacturers’ instructions, and hybridized to the HumanHT-12 v4 Expression BeadChip (Illumina), which contains more than 47,000 probes derived from the NCBI RefSeq Release 38 and other sources, at 58°C for 16 h. The chip was washed, stained with streptavadin-Cy3, and scanned with the Illumina BeadStation 500 and BeadScan. Using the Illumina's GenomeStudio software, we normalized the signals using the “cubic spline algorithm” that assumes that the distribution of transcript abundance is similar in all samples, according to the method proposed by Workman *et al.* [30]. The background signal was removed using the “detection *p*-value algorithm” to remove targets with signal intensities equal or lower than that of irrelevant probes (with no known targets in the human genome but thermodynamically similar to the relevant probes). Common and unique sets of genes and enrichment analysis were performed using the MetaCore Software (Thompson Reuters) as previously reported [14]. The microarray original data have been submitted to Gene Expression Omnibus (GEO) database (Accession number: GSE79548).

### *RNA* interference and plasmid transfection

*ISG15* and *ISG20* ON-TARGET plus SMART pool siRNA, or negative control siRNA (n-siRNA) (Dharmacon), were delivered using the DharmaFECT transfection reagent according to the manufacturer's instructions. For plasmid transfection, PDLF were transfected in 12-well plates with miR-K12-1 recombinant construct or control vector as previously described [28] by using Lipofectamine 3000 (Invitrogen) for 48 h. Transfection efficiency was normalized through co-transfection of a lacZ reporter construct and determination of β-galactosidase activity using a commercial β-galactosidase enzyme assay system according to the manufacturer's instructions (Promega).

### Immunoblotting

Total cell lysates (20 μg) were resolved by 10% SDS–PAGE, transferred to nitrocellulose membranes, and immunoblotted with antibodies for K8.1 (ABI) and β-Actin (Sigma) for loading controls. Immunoreactive bands were identified using an enhanced chemiluminescence reaction (Perkin-Elmer), visualized by autoradiography and quantitated using Image-J software.

### qRT-PCR

Total RNA was isolated using the RNeasy Mini kit (QIAGEN), and cDNA was synthesized from equivalent total RNA using a SuperScript III First-Strand Synthesis SuperMix Kit (Invitrogen) according to the manufacturer's instructions. Primers used for amplification of target genes are displayed in Supplementary Table S1. Amplification was carried out using an iCycler IQ Real-Time PCR Detection System, and cycle threshold (Ct) values were tabulated in duplicate for each gene of interest in each experiment. “No template” (water) controls were used to ensure minimal background contamination. Using mean Ct values tabulated for each gene, and paired Ct values for β-actin as a loading control, fold changes for experimental groups relative to assigned controls were calculated using automated iQ5 2.0 software (Bio-rad). For amplification of viral miRNAs, cDNA was synthesized using the Taqman miRNA RT kit (Applied Biosystems), and qPCR was performed using the Taqman MicroRNA Assays kit (Applied Biosystems) and a 7500 Real Time PCR System. Fold changes for microRNA were calculated using paired Ct values for RNU6B as recommended by the manufacturer (Applied Biosystems).

### Statistical analysis

Significance for differences between experimental and control groups was determined using the two-tailed Student's *t*-test (Microsoft Excel 2010), and *p* values < 0.05 or <0.01 were considered significant or highly significant.

## SUPPLEMENTARY MATERIALS


