# Supplementary material for: Transcriptomic analysis of KSHV-infected primary oral fibroblasts: The role of interferon-induced genes in the latency of oncogenic virus
Source: Oncotarget. 2016 May 30;7(30):47052–60. doi: 10.18632/oncotarget.9720 (PMC5216923; doi:10.18632/oncotarget.9720)
Supplement: Supplementary file 1 [file oncotarget-07-47052-s001.pdf]

Transcriptomic analysis of KSHV-infected primary oral fibroblasts: The role of interferon-induced genes in the latency of oncogenic virus

Supplementary Materials

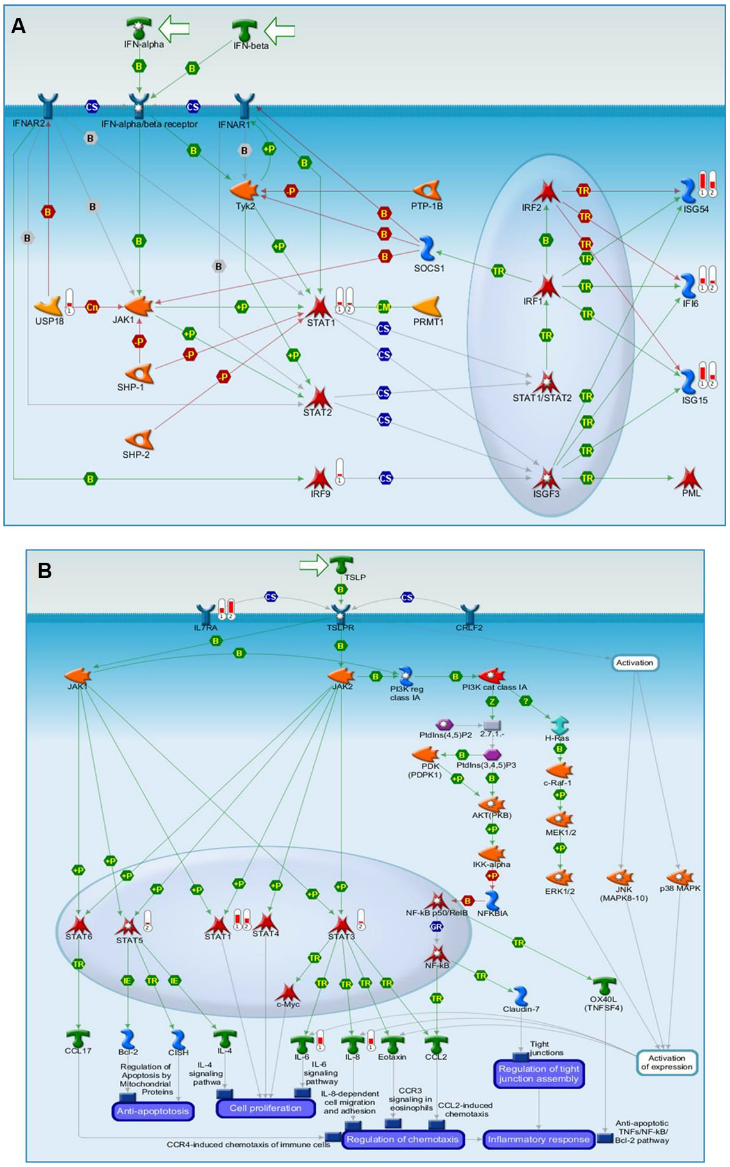

Supplementary Figure S1: The top 2 scored maps (maps with the lowest *p* value) based on the enrichment distribution sorted by 'common' gene set altered in KSHV-infected primary oral fibroblasts. (**A**) Immune response_ IFN α/β signaling pathway. (**B**) Immune response_Thymic stromal lymphopoietin (TSLP) signaling pathway. Experimental data from all files is linked to and visualized on the maps as thermometer like figures. Up-ward thermometers have red color and indicate up-regulated signals and down-ward (blue) ones indicate down-regulated expression levels of the genes.

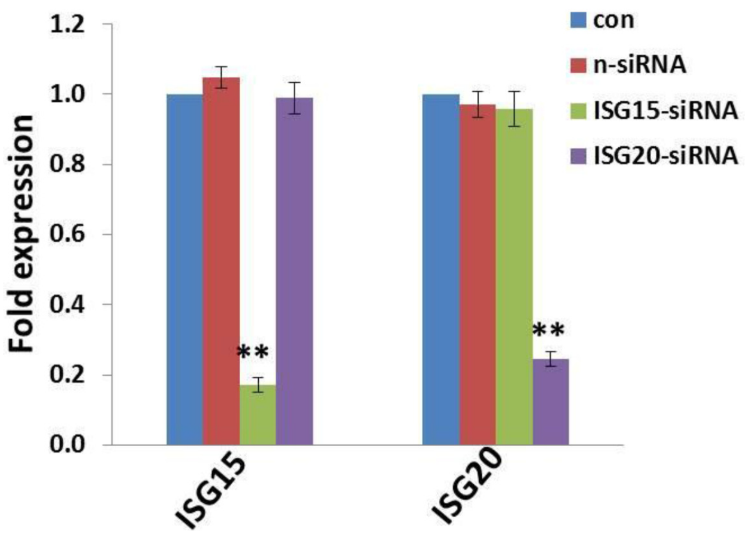

Supplementary Figure S2: Silencing of *ISG15* and/or *ISG20* by RNAi in KSHV-infected primary oral fibroblasts. PDLF were first incubated with purified KSHV (MOI~10) for 2 h, then after 24 h p.i. transfected with either control non-target (n-siRNA), *ISG15*-siRNA or *ISG20*-siRNA for additional 48 h. Gene transcripts were quantified using qRT-PCR. Error bars represent the S.E.M for three independent experiments. ** = $p < 0.01$ (vs n-siRNA group).

Supplementary Table S1: Primer sequences for qRT-PCR in this study

Gene	Sequences (5′→3′)
IFI27	*sense* *GCTACAGTTGTGATTGGAGGAG*<br>*antisense* *AATGGAGCCCAGGATGAA*
IFI44	*sense* *CCTGCCGTTTATTCTGTG*<br>*antisense* *CGTTTACCAACTCCCTTC*
IFIT1	*sense* *CACCCACTTCTGTCTTACT*<br>*antisense* *ACATTCTTGCCAGGTCTA*
IFIT2	*sense* *ATACCAAACAATGCCTACC*<br>*antisense* *GAGCCACAGCGTGTCCTA*
MX1	*sense* *GACATTCGGCTGTTTACC*<br>*antisense* *CTTCCAGTGCCTTGATTT*
MX2	*sense* *ACCGCCATTCGGCACAGT*<br>*antisense* *TGCCCTTGGTTGGCTCCT*
ISG15	*sense* *TGGACAAATGCGACGAACC*<br>*antisense* *CCCGCTCACTTGCTGCTT*
ISG20	*sense* *CCACGGTGCTGTGCTGTA*<br>*antisense* *GCCGCTCATGTCCTCTTT*
LANA	*sense* *TCCCTCTACACTAAACCCAATA*<br>*antisense* *TTGCTAATCTCGTTGTCCC*
RTA	*sense* *TAATGTCAGCGTCCACTCC*<br>*antisense* *TTCTGGCACGGTCAAAGC*
vGPCR	*sense* *CATCCGCTGCACTGTTAA*<br>*antisense* *GCTTTGTCCTCCTCACCA*
K8.1	*sense* *CACCACAGAACTGACCGATG*<br>*antisense* *TGGCACACGGTTACTAGCAC*
β-actin	*sense* *GGAAATCGTGCGTGACATT*<br>*antisense* *GACTCGTCATACTCCTGCTTG*
